# Cystic fibrosis: desensitization in delayed hypersensitivity reactions to elexacaftor/tezacaftor/ivacaftor

**DOI:** 10.3389/fphar.2024.1392986

**Published:** 2024-06-12

**Authors:** L. Gómez-Ganda, P. Galván-Blasco, A. Fernández-Polo, V. Cardona, B. García-Palop, CJ Parramón-Teixidó, E. Polverino, A. Álvarez-Fernández

**Affiliations:** ^1^ Pharmacy Department, Vall d’Hebron University Hospital, Barcelona, Spain; ^2^ Allergology Department, Vall d’Hebron University Hospital, Barcelona, Spain; ^3^ Vall Hebron Institut de Recerca (VHIR), Vall d’Hebron University Hospital, Barcelona, Spain; ^4^ Cystic Fibrosis Unit, Pulmonology Department, Vall d’Hebron University Hospital, Barcelona, Spain

**Keywords:** cystic fibrosis, cystic fibrosis transmembrane conductance regulator modulator, delayed hypersensitivity, desensitization, elexacaftor/tezacaftor/ivacaftor, rash

## Abstract

**Background:** Cystic fibrosis transmembrane conductance regulator modulators are the only available treatment for cystic fibrosis. Although elexacaftor/tezacaftor/ivacaftor (ELX/TEZ/IVA) is well-tolerated, rash has been reported as very frequent. In severe rashes, ELX/TEZ/IVA withdrawal is necessary, leading to clinical deterioration. The objective of the study is to increment the experience of ELX/TEZ/IVA desensitization.

**Methods:** Adult patients who developed a delayed hypersensitivity rash to ELX/TEZ/IVA between December 2021 and February 2023 and required withdrawal due to ineffective rescue medication were included. Skins test for ELX/TEZ/IVA and IVA were conducted to establish hypersensitivity mechanism. Balijepally ELX/TEZ/IVA desensitization protocol was selected. In cases where desensitization had to be discontinued due to rash, an extended desensitization was proposed. Clinical and health-related quality of life parameters were collected before ELX/TEZ/IVA and after desensitization.

**Results:** 162 patients (81 women, 31.2 [23.8–42.5] years) started ELX/TEZ/IVA, developing rash 12 of them (7.4%, six women). Six patients (five women) required stopping ELX/TEZ/IVA and were selected for desensitization. Skin tests indicated delayed type-IV hypersensitivity in one patient. Two patients presented adequate tolerance to desensitization; while, four patients developed rash. Three of these patients, successfully concluded extended desensitization (one patient declined participation). No significant clinical deterioration or quality of life worsening was observed during desensitization; in fact, there was an improvement in practically all mesured parameters. All five patients who resumed ELX/TEZ/IVA are currently receiving therapy with good tolerance.

**Conclusion:** Desensitization to ELX/TEZ/IVA could be a successful and safe strategy for reintroducing this essential treatment in cases of a delayed hypersensitivity rash.

## 1 Introduction

Cystic fibrosis (CF) is the most common rare autosomal recessive disease among Caucasian populations, being a life-limiting condition for patients. It is caused by mutations in the CF transmembrane conductance regulator (CFTR), a protein which encodes for an ion channel responsible for transporting chloride ions across epithelial cells. CFTR alteration causes abnormal mucus secretion and multiorgan dysfunction, including recurrent respiratory infections, airway obstruction, and pancreatic insufficiency. In case of chronic airway impairment, it can lead to progressive lung damage and respiratory failure, and even premature death (Gramegna et al., 2020; Dawood et al., 2022).

Conventional treatment for CF was focused on the management of symptoms resulting from CFTR dysfunction. Nevertheless, the development of CFTR modulators has meant a paradigm change in the prognosis and quality of life for CF patients. CFTR modulators permit, for the first time, the targeted treatment of the molecular consequences of CFTR mutations and the restoration of CFTR protein function and, nowadays, they are the only therapeutic alternative available for the treatment of CF. The most recent CFTR modulators combination, elexacaftor/tezacaftor/ivacaftor (ELX/TEZ/IVA), is authorized for the treatment of CF in patients aged 6 years and older with at least one *Phe508del* CFTR mutation in combination with ivacaftor (IVA). *Phe508del* is the most common worldwide mutation of CFTR, with a prevalence of 80.3% in European population in 2021 (Zolin et al., 2023). Consequently, a significant percentage of patients are eligible for treatment with ELX/TEZ/IVA. ELX/TEZ/IVA has demonstrated to significantly improve lung function, sweat chloride concentration, pulmonary exacerbations, bodyweight, and respiratory-related quality of life (Gramegna et al., 2020; Dawood et al., 2022; Wang et al., 2022).

Due to the impact of ELX/TEZ/IVA on the clinical evolution and quality of life of CF patients, ensuring adequate therapeutic compliance is essential. Although ELX/TEZ/IVA is well-tolerated in most patients, rash has been reported as a very frequent adverse reaction, with a higher incidence in women and, particularly those taking hormonal contraception (Dawood et al., 2022; AEMPS, 2024). Currently, there are no recommendations about rash management, and it depends on its severity. In self-limiting cases, symptomatology management with topical and oral corticoids and antihistamines could be enough. Unfortunately, in cases of severe rash, the only options are the temporal o permanent withdrawal of ELX/TEZ/IVA, which can lead to a significant detrimental impact on the prognosis of patients (Gramegna et al., 2020; Dawood et al., 2022). Hence, a well-designed desensitization protocol to ELX/TEZ/IVA that allows a safe reintroduction of the treatment plays a fundamental role in the clinical evolution of patients. Although desensitization protocols with CFTR modulators have shown successful results, the experience is limited and it is mostly based on case reports.

The aim of this study is to share our results of ELX/TEZ/IVA desensitization in order to increase the data of experience for helping other healthcare professionals in the reintroduction of this essential treatment to CF patients who have presented cutaneous hypersensitivity reactions.

## 2 Materials and methods

Our hospital is a national reference centre for CF patients, staffed with specialized medical and pharmaceutical professionals and equipped with dedicated facilities.

Before starting the study, it was presented to and approved by the hospital’s ethics committee.

The period of study was between December 2021 (date of authorization of ELX/TEZ/IVA in Spain) and February 2023.

### 2.1 Patient database

Biodemographic, clinical, and therapeutic data for all patients who start treatment with ELX/TEZ/IVA are systematically recorded in a database (Microsoft Excel^®^ 2016) according to the protocol established by the Pharmacy Department with the aim of adequately monitoring the safety and adherence to the therapy. During the treatment, any adverse reactions are documented and categorized based on the organ involved and the severity of the reaction. Furthermore, information about the need for dose reduction, treatment withdrawal, or resumption is collected.

### 2.2 Patient selection

Adult patients (aged 18 years or older) who developed a delayed hypersensitivity rash after the initiation of ELX/TEZ/IVA and required withdrawal due to ineffective control of skin symptoms with rescue symptomatic medication were selected for the study. According to the protocol, these patients are referred to the Allergology Department for evaluation. Exclusion criteria included prior severe skin reactions, such as drug rash with eosinophilia and systemic symptoms (DRESS) or Steven-Johnson syndrome. Written informed consent for ELX/TEZ/IVA desensitization was required for patient inclusion in the study.

### 2.3 Skin test

All included patients underwent skin tests with ELX/TEZ/IVA and IVA to determine if a mechanism of selective delayed hypersensitivity could be established. Although there are no standardized protocols or established data regarding the optimal drug concentration for skin tests prepared from oral formulations, the most common practice is to dissolve the tablet in 0.9% saline serum at the highest feasible concentration (Barbaud et al., 2001; Brockow et al., 2013; Barbaud et al., 2022). Therefore, in accordance with the guidelines, we opted for a 10% concentration for the epicutaneous skin test. The skin tests were prepared extemporaneously in the Allergology Department by crushing the ELX/TEZ/IVA and IVA tablets using a completely impurity-free device, with petrolatum as the base excipient. IVA was prepared first, followed by ELX/TEZ/IVA, to prevent cross-contamination between the two preparations. Subsequently, the preparations were incorporated into a patch and applied on the upper back. The first reading was taken at 48 h, followed by a second reading at 96 h. Skin test positivity was considered according to current guidelines, and one healthy control served as a negative control (Brans and Mahler, 2023).

### 2.4 Desensitization protocols

A bibliographic review of reported CFTR modulators desensitization protocols was conducted and Balijepally R *et al.* (Balijepally et al., 2022) protocol was selected because dose escalation was based on the pharmacokinetics of ELX/TEZ/IVA and IVA. According to this protocol, the patient receives ELX/TEZ/IVA in combination with IVA, and the dose of both is escalated weekly, as is shown in Table 1.

**TABLE 1:** Elexacaftor/tezacaftor/ivacaftor desensitization protocol (Balijepally et al., 2022).

Week	ELX/TEZ/IVA (morning)	IVA (evening)
Week 1	0.25 tablet (25 mg/12.5 mg/18.75 mg)	0.25 tablet (37.5 mg)
Week 2	0.5 tablet (50 mg/25 mg/37.5 mg)	0.5 tablet (75 mg)
Week 3	0.75 tablet (75 mg/37.5 mg/56.25 mg)	0.75 tablet (112.5 mg)
Week 4	1 tablet (100 mg/50 mg/75 mg)	1 tablet (150 mg)
Week 5	1.25 tablet (125 mg/62.5 mg/93.75 mg)	1 tablet (150 mg)
Week 6	1.5 tablet (150 mg/75 mg/112.5 mg)	1 tablet (150 mg)
Week 7	1.75 tablet (175 mg/87.5 mg/131.25 mg)	1 tablet (150 mg)
Week 8	2 tablets (200 mg/100 mg/150 mg)	1 tablet (150 mg)

ELX/TEZ/IVA: elexacaftor/tezacaftor/ivacaftor, IVA: ivacaftor.

ELX/TEZ/IVA, commercialized tablet: 100 mg/50 mg/75 mg. IVA, commercialized tablet: 150 mg.

To ensure an adequate and precise dosage, personalized dose capsules of ELX/TEZ/IVA and IVA were prepared for each patient in the Pharmacy Department, using the commercial presentations of each drug and microcrystalline cellulose as a diluent excipient. Due to the importance of adequate compliance to the desensitization protocol, pharmaceutical care visits were scheduled for the first dispensation and subsequent ones.

All patients were treated with concomitant oral antihistamines during the desensitization protocol, with discontinuation two weeks after reaching the standard dosage. Patients were instructed to promptly contact the Allergology Department within 24 h if they experienced any abnormal symptoms, especially involving the skin, and were advised not to continue with the desensitization protocol until an evaluation had been performed.

Given the potential association between rash and the use of concomitant hormonal contraceptives, it was decided to discontinue oral contraceptive therapy at least one month before starting the ELX/TEZ/IVA and IVA desensitization.

In case of rash during the desensitization and a need to discontinue the treatment, an extended desensitization protocol was proposed. In contrast to the previous desensitization protocol, the extended desensitization protocol is initiated with ELX/TEZ/IVA in monotherapy at a lower dosage, introducing IVA from week eight onwards, and carrying out a slower weekly increment of dosage (Table 2).

**TABLE 2:** Extended elexacaftor/tezacaftor/ivacaftor desensitization protocol.

Week	ELX/TEZ/IVA (morning)	IVA (evening)
Week 1	0.10 tablet (10 mg/5 mg/7.5 mg)	-
Week 2	0.2 tablet (20 mg/10 mg/15 mg)	-
Week 3	0.4 tablet (40 mg/20 mg/30 mg)	-
Week 4	0.75 tablet (75 mg/37.5 mg/56.25 mg)	-
Week 5	1 tablet (100 mg/50 mg/75 mg)	-
Week 6	1.5 tablet (150 mg/75 mg/112.5 mg)	-
Week 7	2 tablets (200 mg/100 mg/150 mg)	-
Week 8	2 tablets (200 mg/100 mg/150 mg)	0.25 tablet (37.5 mg)
Week 9	2 tablets (200 mg/100 mg/150 mg)	0.5 tablet (75 mg)
Week 10	2 tablets (200 mg/100 mg/150 mg)	0.75 tablet (112.5 mg)
Week 11	2 tablets (200 mg/100 mg/150 mg)	1 tablet (150 mg)

ELX/TEZ/IVA: elexacaftor/tezacaftor/ivacaftor, IVA: ivacaftor.

ELX/TEZ/IVA, commercialized tablet: 100 mg/50 mg/75 mg. IVA, commercialized tablet: 150 mg.

### 2.5 Clinical and health-related quality of life follow-up parameters

In order to assess that the desensitization protocol did not lead to clinical deterioration due to the lower dose of ELX/TEZ/IVA and IVA, forced vital capacity (FVC), forced expiratory volume in one second (FEV1), sweat chloride concentration, and body mass index (BMI) were assessed before the start of the treatment and at the end of ELX/TEZ/IVA and IVA desensitization protocol.

Likewise, the health-related quality of life of the patients was evaluated before the start of treatment and after ELX/TEZ/IVA and IVA desensitization protocol, using the Cystic Fibrosis Questionnaire-Revised (CFQ-R). CFQ-R is a disease-specific health-related quality of life measure for children, adolescents and adults with CF, that includes several different domains (physical, vitality, emotion, eating, treat, health, social, body, role, weight, respiratory and digestive), giving a score on a 0–100 scale with higher scores indicating better quality of life (Quittner et al., 2005).

As the sample size was insufficient to presume the normality in the data and neither graphically evaluation and skewness/kurtosis tests allow a correct assessment of normality, the Fisher-Pitman permutation test was performed to compare the change before and after desensitization. Statistical comparisons were performed using StataCorp. 2019 (Stata Statistical Software: Release 16. College Station, TX: StataCorp LLC.).

## 3 Results

During the study period, a total of 162 adult patients initiated ELX/TEZ/IVA treatment at our hospital, 81 (50%) patients were female, and the median age was 31.2 years (interquartile range: 23.8–42.5).

Twelve patients (7.4%)—six women and six men-presented some form of skin adverse reaction at the onset of ELX/TEZ/IVA treatment. In six of the cases, the adverse reaction was resolved without the need to discontinue treatment. However, the other six patients required treatment discontinuation and were subsequently selected for ELX/TEZ/IVA desensitization, with their written informed consent obtained before their inclusion in the study (Table 3). The patients selected were predominantly women (five women and one man), aged between 22 and 55 years. In all cases, the reaction appeared between the first and the second week of treatment, affecting the trunk and proximal region of the extremities. The rashes were defined as progressively confluent maculopapular. None of the patients presented severe signs such as high fever, lymphadenopathy, mucosal involvement, or a positive Nikolsky sign, and laboratory analyses showed no abnormalities. Five of these patients experienced resolution of their rashes within 10 days following the discontinuation of ELX/TEZ/IVA and the initiation of treatment with oral antihistamines and corticosteroids.

**TABLE 3:** Biodemographic, clinical, therapeutic and skin reaction data of patients.

Patient	Sex	Age (years)	CF mutation	Previous therapy with CFTR modulator	Concomitant hormonal contraceptive treatment	Day of skin reaction since ELX/TEZ/IVA start	Time until resolution
Patient 1	F	30	*F508del/F508del*	Yes	No	7 days	5 weeks
Patient 2	F	22	*F508del/D1152H*	No	Yes	7 days	7 days
Patient 3	F	53	*F508del/P205S*	No	No	14 days	7 days
Patient 4	F	34	*F508del/P205S*	No	Yes	7 days	7 days
Patient 5	M	55	*F508del/R117H*	No	No	10 days	9 days
Patient 6	F	44	*DF508/R352W*	No	No	14 days	24 h

*CF: cystic fibrosis, CFTR: cystic fibrosis transmembrane conductance regulator,* ELX/TEZ/IVA: elexacaftor/tezacaftor/ivacaftor, *F: female, M: male*.

### 3.1 Skin tests

Epicutaneous skin tests with ELX/TEZ/IVA and IVA, conducted at 48 h and 96 h, resulted a positive result only in patient 4, indicating a delayed type IV hypersensitivity reaction. The skin tests for the healthy control were negative for both intervals skin tests.

### 3.2 Desensitization procedure

All patients initiated taking oral antihistamines 72 h prior to starting the ELX/TEZ/IVA and IVA desensitization protocol. Patients 2 and 4 had discontinued hormonal contraceptive treatment at least one month before initiating the desensitization.

Patients 1 and 2 demonstrated correct tolerance to the first desensitization protocol; however, patients 3 and 4 developed a skin rash in the proximal inferior extremities and trunk 8 h after receiving the first dose of ELX/TEZ/IVA, and patient 5 after 12 h after the first dose. Patient 6 experienced a pruritic macular rash five days after the second increase in increment of ELX/TEZ/IVA dose, which initially appeared in the proximal region of the lower extremities and later spread to the trunk.

According to the protocol, patients reported any adverse reactions to the Allergology Department within 24 h of the onset of skin symptoms, after which they discontinued ELX/TEZ/IVA. In all cases, doses of antihistamine were increased, and only patient 5 required the addition of oral corticosteroids due to an inadequate response to antihistamines. Patients were reassessed weekly, and once the rash was resolved, they were considered for the extended desensitization protocol (Table 2). Patients 3, 5, and 6 underwent the extended desensitization protocol and presented successful tolerance. Nevertheless, patient 4 declined the extended desensitization protocol for personal reasons, and permanently discontinued treatment.

Except for patient 4, all patients successfully resumed ELX/TEZ/IVA and are currently on therapy without any reports of rash, with a median follow-up time of 14.9 months (interquartile range: 12.5–16.7).

### 3.3 Clinical and health-related quality of life follow-up parameters

**FIGURE 1:**
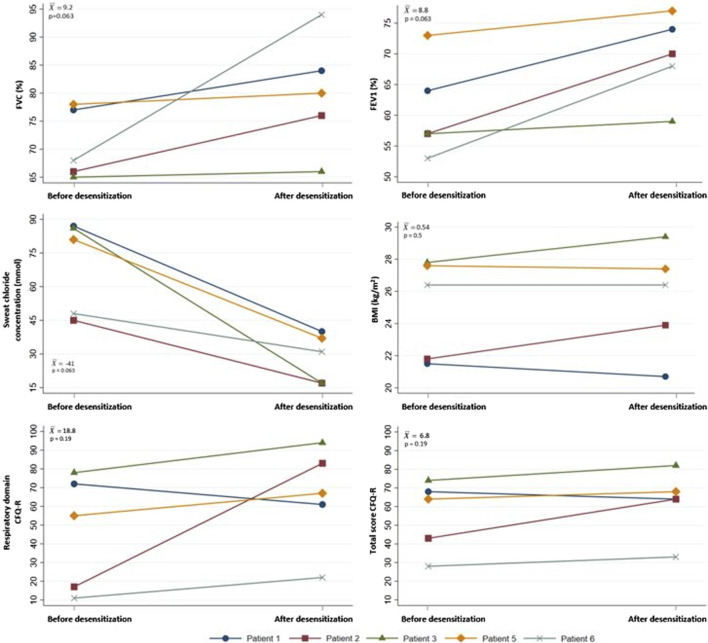
Changes in clinical parameters and CFQ-R before the start of treatment with ELX/TEZ/IVA and IVA and after completing the ELX/TEZ/IVA and IVA desensitization protocol. BMI: body mass index, CFQ-R: Cystic Fibrosis Quesionnaire-Revised, ELX/TEZ/IVA: elexacaftor/tezacaftor/ivacaftor, FEV1: forced expiratory volume in one second, FVC: forced vital capacity, $\bar{x}$ : mean change, p: *p*-value of Fisher-Pitman permutation test.

The clinical parameters FVC, FEV1, sweat chloride concentration, and BMI, along with the health-related quality of life parameter CFQ-R, before the initiation of treatment and after completing the ELX/TEZ/IVA and IVA desensitization protocol, are shown in Figure 1. Since patient 4 did not complete the desensitization protocol, the follow-up data for clinical and quality of life parameters were considered not valid and was excluded from this section of the results. In case of patients 5 and 6, the data were collected one and two weeks, respectively, before finishing the desensitization protocol and receiving the full dose.

## 4 Discussion

The recent development and commercialization of CFTR modulators have significantly improved the survival and quality of life of CF patients, by targeting the molecular consequences of CFTR mutations and restoring CFTR protein function. The triple combination of ELX/TEZ/IVA, used in combination with IVA, provides a therapeutic option for patients with at least one *Phe508del* CFTR mutation, the most prevalent CFTR mutation worldwide (Gramegna et al., 2020; Leonhardt et al., 2021; Dawood et al., 2022; Wang et al., 2022).

Given that CFTR modulators represent the only therapeutic alternative available for CF patients, the withdrawal of ELX/TEZ/IVA due to adverse reactions can have detrimental effects on their prognosis. Even though ELX/TEZ/IVA has shown a favourable safety profile, rash is classified as a very frequent adverse reaction (Dawood et al., 2022; AEMPS, 2024). Clinical studies have reported a rash incidence of 10.9%, with a higher occurrence in women (16.3%) than in men (5.8%). Furthermore, the incidence of rash was superior (20.5%) among women who were using hormonal contraception (AEMPS, 2024). The incidence of rash in our study was lower than previously reported, with a similar distribution between the sexes (AEMPS, 2024). However, the majority of patients who needed to discontinue ELX/TEZ/IVA and undergo the desensitization protocol were mostly women.

In cases of severe rash, symptomatology management alone becomes insufficient, and the only options are temporary or permanent withdrawal of the therapy (Gramegna et al., 2020; Dawood et al., 2022). An adequate desensitization protocol to CFTR modulators is an essential option for the safe reintroduction of treatment and to ensure appropriate clinical evolution of the patient.

Leonhardt et al., 2021 reported on a female patient who developed a diffuse rash with papules seven days after starting ELX/TEZ/IVA treatment. Due to the severity of rash, treatment was discontinued and supportive treatment was administered. However, because of a decline in lung function, two different desensitization protocols to ELX/TEZ/IVA were carried out, with the patient showing good tolerance to the second. (Cheng et al., 2022) published a case of a male, who, also, required two desensitization protocols to ELX/TEZ/IVA for a successful reintroduction of treatment after developing a severe widespread rash with fever developed nine days after starting the therapy.

Balijepally et al., 2022 described successful results using a slow desensitization protocol for two female patients who developed delayed, spread, pruritic and follicular rashes to ELX/TEZ/IVA, requiring treatment withdrawal. In these cases, the desensitization protocols were established based on drug pharmacokinetics, the type of hypersensitivity, and patient history. The first patient experienced the rash three weeks after starting therapy, while the second case, who switched from TEZ/IVA to ELX/TEZ/IVA, developed the rash six days after treatment initiation. Both cases were diagnosed with type IV hypersensitivity reactions to ELX/TEZ/IVA.

Another case was published by Muirhead et al., 2022, a woman who developed rash during the fifth week of treatment with ELX/TEZ/IVA and whom CFTR modulator therapy was successfully reintroduced after desensitization.

Only two paediatric cases have been reported, both showing favourable results from desensitization protocols for ELX/TEZ/IVA after treatment was discontinued due torash. One patient presented the rash on the first day of treatment, while the other one developed after the third dose of ELX/TEZ/IVA (Diseroad et al., 2022; Loyd et al., 2022).

Recently, Ali et al., 2023 published a case involving a female who successfully underwent a rapid desensitization protocol for ELX/TEZ/IVA after discounting CFTR treatment due to several episodes of pruritic and urticarial rash.

In all reported cases, as well as in all patients in our study, discontinuation of ELX/TEZ/IVA resulted in a decline in lung function and a deterioration in the clinical status of the patients. The desensitization protocols allowed an effective and a safe reintroduction of the treatment, leading to significant improvements in lung function and clinical outcomes.

To the best of our knowledge, this study represents the largest reported cohort of patients with ELX/TEZ/IVA-induced delayed hypersensitivity rash who successfully resumed therapy after an oral desensitization protocol. For this reason, we believe that our findings could be extremely helpful for other healthcare professionals in managing the rash and reintroducing ELX/TEZ/IVA treatment.

According to published cases, rash usually appears within the first 10 days of initiating ELX/TEZ/IVA treatment. Our results align with these findings, as the rash was developed during the first two weeks of treatment. Therefore, close monitoring during this initial period is fundamental. Furthermore, it is crucial to provide patients with adequate and comprehensive information about the possible development of a rash and the appropriate actions to take if it occurs.

In contrast to the cases previously cited, this study includes a skin test to assess hypersensitivity to ELX/TEZ/IVA and IVA. However, since ELX/TEZ/IVA and IVA are only available in tablet form, the sensitivity of the test may be compromised by factors such as the low solubility of IVA (Fohner et al., 2017). While a negative patch test does not conclusively rule out drug hypersensitivity, it does provide an initial estimate of the usefulness of skin tests for ELX/TEZ/IVA and IVA. One patient had positive patch tests to ELX/TEZ/IVA and IVA, confirming a type IV hypersensitivity, which aligns with findings reported by Balijepally et al., 2022. Recently, Mir-Ihara et al., 2023 have reported a case where a man presented a maculopapular rash and eosinophilia seven days into treatment with ELX/TEZ/IVA and underwent epicutaneous patch tests with ELX/TEZ/IVA and IVA in 30% petrolatum, showing negative readings at 48, 72, and 96 h.

Except for patient 4, the desensitization protocol has allowed patients to receive the only currently available therapy for treating CF. No clinical deterioration or decline in quality of life was observed in any case during the desensitization; in fact, there was an improvement in practically all measured parameters for all patients. Presently, all five patients continue the treatment without any reported skin reactions.

While the statistical potency of the analysis is limited by the sample size, notable improvements in both individual and average clinical outcomes and quality of life have been observed. We believe that these results could achieve statistical significance with larger patient cohorts.

We would like to emphasize that using personalized dose capsules of ELX/TEZ/IVA and IVA to ensure an individualized and precise dosing, along with multidisciplinary, closed monitoring during desensitization, is crucial for achieving successful and safe outcomes. Moreover, given the higher reported incidence of rash among women using hormonal contraception, we consider that discontinuation of this therapy is recommendable, at least during the desensitization period.

In conclusion, a well-established desensitization protocol for ELX/TEZ/IVA could be a successful and safe strategy for CF patients who develop a delayed hypersensitivity rash as an adverse reaction to ELX/TEZ/IVA, allowing for the reintroduction of this essential treatment in this patient population.

## Data Availability

The raw data supporting the conclusion of this article will be made available by the authors, without undue reservation.

## Abbreviations

BMI, body mass index; CF, cystic fibrosis; CFQ-R, Cystic Fibrosis Questionnaire-Revised; CFTR, cystic fibrosis transmembrane conductance regulator; ELX/TEZ/IVA, elexacaftor/tezacaftor/ivacaftor; FEV1, forced expiratory volume in one second; FVC, forced vital capacity, IVA, ivacaftor.
